# Arc Interacts with the Integral Endoplasmic Reticulum Protein, Calnexin

**DOI:** 10.3389/fncel.2017.00294

**Published:** 2017-09-20

**Authors:** Craig Myrum, Jonathan Soulé, Margarethe Bittins, Kyle Cavagnini, Kevin Goff, Silje K. Ziemek, Maria S. Eriksen, Sudarshan Patil, Adrian Szum, Rajeevkumar R. Nair, Clive R. Bramham

**Affiliations:** ^1^Dr. Einar Martens Research Group for Biological Psychiatry, Center for Medical Genetics and Molecular Medicine, Haukeland University Hospital Bergen, Norway; ^2^Department of Biomedicine and the K.G. Jebsen Center for Research on Neuropsychiatric Disorders, University of Bergen Bergen, Norway

**Keywords:** arc, calnexin, endoplasmic reticulum, proximity ligation assay, endocytosis, synaptic plasticity

## Abstract

Activity-regulated cytoskeleton-associated protein, Arc, is a major regulator of long-term synaptic plasticity and memory formation. Here we reveal a novel interaction partner of Arc, a resident endoplasmic reticulum transmembrane protein, calnexin. We show an interaction between recombinantly-expressed GST-tagged Arc and endogenous calnexin in HEK293, SH-SY5Y neuroblastoma and PC12 cells. The interaction was dependent on the central linker region of the Arc protein that is also required for endocytosis of AMPA-type glutamate receptors. High-resolution proximity-ligation assays (PLAs) demonstrate molecular proximity of endogenous Arc with the cytosolic C-terminus, but not the lumenal N-terminus of calnexin. In hippocampal neuronal cultures treated with brain-derived neurotrophic factor (BDNF), Arc interacted with calnexin in the perinuclear cytoplasm and dendritic shaft. Arc also interacted with C-terminal calnexin in the adult rat dentate gyrus (DG). After induction of long-term potentiation (LTP) in the perforant path projection to the DG of adult anesthetized rats, enhanced interaction between Arc and calnexin was obtained in the dentate granule cell layer (GCL). Although Arc and calnexin are both implicated in the regulation of receptor endocytosis, no modulation of endocytosis was detected in transferrin uptake assays. Previous work showed that Arc interacts with multiple protein partners to regulate synaptic transmission and nuclear signaling. The identification of calnexin as a binding partner further supports the role of Arc as a hub protein and extends the range of Arc function to the endoplasmic reticulum, though the function of the Arc/calnexin interaction remains to be defined.

## Introduction

Activity-regulated cytoskeleton-associated protein (Arc) has been established as a major regulator of protein synthesis-dependent synaptic plasticity (Bramham et al., 2010; Korb and Finkbeiner, 2011; Shepherd and Bear, 2011; Nikolaienko et al., 2017a). Arc synthesis is essential for long-term forms of synaptic plasticity such as long-term potentiation (LTP), long-term depression (LTD; Park et al., 2008; Waung et al., 2008) and homeostatic scaling (Shepherd et al., 2006). These Arc-dependent changes in synaptic efficacy are thought to be important in a wide range of behavioral adaptations including long-term memory formation (Plath et al., 2006; Minatohara et al., 2016).

As an immediate-early gene, basal Arc mRNA levels are low but rapidly peak following learning-related stimuli or induction of LTP by high-frequency stimulation (HFS) of afferents. Arc is tightly regulated at several levels including mRNA transport and degradation (Steward et al., 1998; Giorgi et al., 2007), translation (Bloomer et al., 2008; Panja et al., 2009; Panja and Bramham, 2014), ubiquitination (Greer et al., 2010; Soulé et al., 2012; Mabb et al., 2014), SUMOylation (Craig et al., 2012; Nair et al., 2017) and phosphorylation (Gozdz et al., 2017; Nikolaienko et al., 2017b).

The molecular role of Arc protein is intriguingly diverse, likely owing to its flexible, hub-like properties (Myrum et al., 2015). Arc is localized to neuronal synapses, but is also found in the dendritic shaft, neuronal soma and the nucleus. At the synapse, Arc has been shown to regulate endosomal trafficking of AMPA-type glutamate receptors, presumably by regulating dynamin GTPase activity (Chowdhury et al., 2006; Byers et al., 2015; DaSilva et al., 2016). By binding presenilin 1, Arc is also involved in the endosomal trafficking and proteolytic processing of amyloid precursor protein and Notch1 (Alberi et al., 2011; Wu et al., 2011), which suggest a governing role of Arc in regulating protein sorting. Arc expression is also critical for the stabilization of F-actin at activated synapses during LTP at activated synapses (Messaoudi et al., 2007), it complexes with the actin-binding protein Drebrin A (Messaoudi et al., 2007; Nair et al., 2017) and affects spine density and morphology (Peebles et al., 2010). Finally, Arc can regulate synaptic strength at the level of gene expression by localizing to nuclear promyelocytic leukemia bodies and controlling expression of the AMPA-type glutamate receptors (Bloomer et al., 2007; Korb et al., 2013).

Despite Arc’s key role in memory consolidation processes and enduring forms of synaptic plasticity, the molecular mechanisms by which it carries these actions out remain unclear. To shed light on the molecular context of these mechanisms, we sought to identify novel Arc binding partners. Using a GST-based pull-down assay and mass spectrometry, we found that Arc interacts with the cytosolic domain of the transmembrane endoplasmic reticulum (ER) protein, calnexin. The interaction was confirmed by immunoprecipitations (IPs) and proximity-ligation assays (PLAs), both *in vitro* and *in vivo*. Lastly, we also begin to explore the possible functional role that Arc may play at the surface of the ER.

## Materials and Methods

### Construction, Expression and Purification of GST-Arc

The Arc coding sequence of rat origin plus a C-terminal histidine tag was inserted into the multiple cloning site of the pGEX plasmid and the sequence was analyzed by DNA sequencing. The plasmid was transformed into BL21 (DE3) CodonPlus competent cells and plated on an agar plate containing 100 μg/ml ampicillin and 34 μg/mL chloramphenicol. A 10 mL LB starter culture was inoculated, grown overnight and diluted in 500 ml LB containing 100 μg/mL ampicillin and 34 μg/ml chloramphenicol in a bacterial shaker at 250 rpm at 37°C until OD600 = 0.6. Isopropyl β-D-1-thiogalactopyranoside (IPTG) was added to a final concentration of 0.6 mM and incubated for an additional 4 h. The culture was at 4°C for 45 min at 4000 rpm. The supernatant was removed, the pellet was resuspended in lysis buffer (pH 8, 1 M phosphate buffer, 500 mM NaCl, 1 mM PMSF, 1× Roche Protease Inhibitor, 1% Triton-X100, 10 mM imidazole, 0.3% Sarkosyl, 10 mM 2-mercaptoethanol, 5% glycerol), sonicated on ice 3 × 5 s with 1 min interval. The sample was centrifuged at 4°C for 20 min at 14,000 rpm and the supernatant was collected. A Qiagen Ni-NTA column was loaded with 600 μL of equilibration buffer (pH 8, 0.1 M phosphate buffer, 500 mM NaCl, 0.5% Triton-X100, 10 mM imidazole) and spun down at 4°C, 700 g for 4 min. Flow-through was discarded and the column was reloaded with 600 μL of crude GST-Arc lysate and spun down at 4°C, 700 g for 4 min. Flow-through was reloaded 2× on the same column and spun down at 4°C, 700 g for 4 min each time. The column was then washed 3× with 600 μL wash buffer (equilibration buffer with 40 mM imidazole) and spun down at 4°C, 700 g for 4 min. A new collection tube was attached to the column, 200 μL elution buffer (pH 8, 0.1 M phosphate buffer, 50 mM NaCl, 200 mM imidazole, 1.0 mM PMSF, 1× Roche protease inhibitor, 1.0 mM DTT) was added, and Arc-GST was eluted by centrifugation at 4°C, 700 g for 4 min. One additional fraction was eluted in 200 μl elution buffer. Protein concentration was measured with a Nanodrop spectrophotometer.

### Cell Line Cultures

SH-SY5Y cells (human), HEK293 cells (human) and PC12 cells (rat) were grown in Dulbecco’s modified Eagle medium (DMEM; Sigma) supplemented with 10% fetal bovine serum, penicillin/streptomycin and L-glutamine. Cells were seeded in 97 mm petri dishes or 6-well plates (Nunclon). Cells were lysed in PBS containing 0.1% Triton-X100, 1 mM PMSF and Roche Complete Protease Inhibitor Cocktail. The sample was centrifuged at 4°C for 20 min at 14,000 rpm, the supernatant was collected and protein concentration was determined by bicinchoninic acid assay (BCA assay; Thermo Scientific Pierce).

### GST-Affinity Purification

HEK293 cell lysate containing 200 mg total protein and 5 mM DTT was mixed with 30 μL glutathione sepharose 4B (GE-Healthcare/VWR 17-0756-01) and placed on a rotor for 1 h at 4°C. The supernatant was collected and split into two fractions. One fraction was incubated with glutathione beads loaded with GST or GST-GFP. The other fraction was incubated with beads loaded with GST-Arc, rotating at 4°C overnight. Beads were washed 4× with ice-cold PBS and spun down at 800 rpm. Samples were run on a 10% SDS-PAGE gel, stained for 3 h at room temperature (RT) with 0.25% Coomassie brilliant blue dye R-250 (Bio Rad), and destained overnight at RT in destaining solution.

### In-Gel Digestion and Mass Spectrometry

Unidentified gel bands were cut into 1.0 mm cubes. Pieces were washed in 100 μL wash solution at RT for 20 min with slow agitation. The supernatant was discarded and the wash was repeated. Gel pieces were then dried in a vacuumed Rotavapor until dry and 50 μL of 10 mM DTT was added and incubated at 56°C for 45 min. Samples were cooled, DTT was removed, and 50 μL of 55 mM iodoacetamide was added and incubated in the dark at RT for 30 min. Iodoacetamide was removed and washed twice, as earlier described. Gel pieces were dried in a vacuumed Rotavapor. To digest proteins, 30 μL of 3 ng/μL trypsin from porsine were added to each sample and rehydrated on ice for 30 min. Samples were incubated for 16 h at 37°C with slow agitation and then cooled and centrifuged at 1300 rpm for 2 min. The supernatant was saved as the first extraction. To extract peptides, 50 μL of 1% trifluoracetic acid was added and incubated at RT for 20 min with gentle agitation. The supernatant was added to the first extraction. Fifty microliter of 60% ACN/0.1% TFA was added to the gel samples and incubated for 20 min with gentle agitation. The supernatant was pooled with the first two extractions. Extractions were then dried in the vacuumed Rotavapor until 10–15 μL sample remained.

Samples were then concentrated by STop And Go Extraction (STAGE) tips. To make a STAGE tip, a Hamilton needle (22 gauge) with a liquid chromatography tip was pressed against an Empore 3M Extraction Disk to wedge a small piece in the opening. The needle tip was placed into a 20 μL gel-loader tip. Using 0.37 mm diameter capillary tubing, the piece of Empore disk was pushed and packed into the gel-loader tip, forming the column to be used for peptide concentration. The gel-loader tip was cut 2–5 mm below this column. The column was washed by slowly pushing 10 μL MeOH through the tip with a syringe. The column was “wetted” with 10 μL 60% CAN/0.1% TFA, knocked down by flicking the gel-loader tip, and 70% was pushed out. The column was “conditioned” with 10 μL 0.1% TFA, knocked down and 70% was pushed out. Ten microliter of the sample was then added to the column, knocked down and 70% was pushed out. The sample was washed with 0.1% TFA and then dried by pushing air through the column. The sample was eluted directly on a MALDI plate with 1.0 μL alpha-cyano-4-hydroxycinnamic acid (CHCA) matrix containing 50% CHCA-1 (60% CAN and 0.2% TFA) and 50% CHCA-2 (ACN:MeOH:H_2_O; 60:30:10). A peptide calibration standard was also plated adjacent to eluted samples. Samples were analyzed by MALDI-ToF-MS UltraFlex instrumentation and Mascot was used to identify peptides.

### Arc Deletion Mutants, GFP-Arc and mCherry-Calnexin

Arc cDNA residues 1–396 of rat origin were amplified with HindIII and BamHI sites and ligated into the pEYFP-C1 vector (Clontech). This full-length EYFP-tagged Arc was used to amplify various regions of the Arc sequence (see “Results” Section). Suitable primers were used to yield a PCR product with HindIII and BamHI sites, which were also ligated into pEYFP-C1. The calnexin signal sequence required for ER localization was inserted upstream of the mCherry fluorophore and the C-tail was ligated into the multiple cloning site of mCherry-C1. Arc cDNA of human origin was inserted into the multiple cloning site of EGFP-C1. Constructs were checked by DNA sequencing by the dideoxy chain termination method in an automated DNA sequencer (ABI Prism 310).

### Arc Deletion Mutant Transfection and Immunoprecipitation (IP)

HEK293 cells at ~80% confluency in 100 mm Petri dishes were transfected with 8 μg Arc-EYFP plasmid deletion mutants, using 30 μL Lipofectamine 2000 reagent (Invitrogen) according to the manufacturer’s protocol. After 24 h, cells were washed, lysed in lysis buffer, scraped off and centrifuged. To IP calnexin, Protein G Sepharose 4 Fast Flow beads (GE Healthcare) were washed in PBS and then incubated with 2.5 μg calnexin C-20 antibody (Table 1) for 1 h at RT. Beads were washed 3× in lysis buffer and incubated with 500 μg total protein overnight at 4°C. Bound fractions were washed 3× in lysis buffer and analyzed by western blot with GFP antibody (Table 1). A western blot of the input fractions ensured that equal amounts of each yellow fluorescent protein (YFP-Arc) construct were used in each IP.

**Table 1:** Antibodies used in this study (IF, immunofluorescence; IP, immunoprecipitation; WB, western blot).

Antibody	Application	Source	Research resource identifier
Mouse anti-Arc	IF1:200	Santa Cruz sc-17839	AB_626696
Rabbit anti-Arc	IF1:1000	Synaptic Systems 156003	AB_887694
Goat anti calnexin	IP	Santa Cruz C-20	AB_2069146
Rabbit anti-calnexin N-term	IF1:100	Enzo Life Sciences ADI-SPA-865	AB_10618434
Rabbit anti-calnexin C-term	IF1:100	Enzo Life Sciences ADI-SPA-860	AB_10616095
Mouse anti-calnexin C-term	IF1:200	Abnova M08	AB_1137121
Rabbit anti-GFP	WB 0.5 μg/ml	BioVision	AB_222261
Goat anti-mouse Alexa Fluor 568	IF 1:500	Invitrogen A-11031	AB_144696
Goat anti-rabbit Alexa Fluor 647	IF 1:500	Invitrogen A-21245	AB_141775

### SDS-PAGE and Western Blotting

Samples were separated by 10% SDS-PAGE and transferred onto a nitrocellulose membrane (Hybond-C; Amersham). Membranes were blocked for 1 h at RT in Tris-buffered saline/0.1% Tween-20 (TBST) and 3% non-fat dry milk. Primary antibodies were diluted in blocking buffer containing TBST and 5% BSA and applied on membranes overnight at 4°C with constant shaking. Following three washes with TBST, blots were incubated for 1 h at RT in horseradish peroxidase-conjugated secondary antibody diluted in TBST. Blots were then visualized using enhanced chemiluminescence (ECL Western Blotting Substrate; Pierce).

### Primary Hippocampal Cell Culture

Hippocampi were dissected from E18 rat embryos (Wistar RjHan:WI, Janvier labs, France), trypsinized, and plated at 30,000 cells/cm^2^ on Nr.1 glass coverslips coated with poly-D-lysine (Sigma P6407). Cells were cultured in minimal essential medium (Gibco 21430), supplemented with B27 (Gibco 17504), antibiotics (Sigma-Aldrich P4083), 2 mM glutamine, 0.6% glucose and 1 mM pyruvate, and conditioned on astrocyte cultures. Where indicated, cells were treated with 100 ng/mL human BDNF diluted in culture medium (Alomone labs; B-250) for 4 h prior to fixation.

### Immunofluorescence of Primary Cell Culture

Cells were washed twice with PBS, fixed for 10 min with 4% paraformaldehyde/4% sucrose/PBS, washed with PBS, permeabilized 10 min with 0.2% Triton-X-100/PBS, washed with 50 mM NH_4_Cl/PBS, washed with PBS, and blocked for 1 h with 1% Roche Blocking Reagent (Roche 11096176001). Primary antibodies (Table 1) were diluted in blocking buffer. Coverslips with neurons were then inverted onto a drop of antibody solution on parafilm in a humidified chamber at 4°C overnight. Coverslips were washed with PBS, then incubated on a drop of secondary antibody (Table 1) in blocking buffer for 1 h at RT, washed again with PBS, dipped in water and mounted with ProLongGold Antifade Reagent containing DAPI (Invitrogen).

### *In Vivo* Electrophysiology

Adult wild type male Sprague-Dawley rats (180–250 g; NTac:SD; Taconic, Denmark) were anesthetized with urethane (i.p. 1.5 g/kg). Rats were placed in a stereotaxic frame and body temperature was maintained at 37°C throughout the experiment. A bipolar stimulation electrode (NE-200; 0.5 mm tip separation; Rhodes Medical Instruments, Wood hills, CA, USA) was positioned ipsilaterally into the perforant path (7.9 mm posterior to Bregma, 4.2 mm lateral to midline and 2.5 mm ventral from the brain surface). Evoked potential was measured by positioning an insulated tungsten recording electrode (0.075 mm; A-M Systems) in the dentate gyrus (DG; 3.9 mm caudal to Bregma, 2.3 mm lateral to the midline and 2.5–3.3 mm ventral from the brain surface). The recording electrode was lowered into the brain in 0.1 mm increments while monitoring the laminar profile of the response waveform evoked by a 300–400 μA test pulse stimulus. Following 20 min of baseline recording, HFS was applied that consisted of 400 Hz, 8-pulse stimulus trains repeated four times with 10 s between each train. HFS was applied three times with 5 min between each session. Total HFS duration was 10.5 min and the total pulse number was 96 (pulse-width was 0.15 ms). The stimulus intensity used for HFS was twice of that used for test pulses. Evoked responses were recorded for 120 min after HFS. Changes in the fEPSP slope were expressed in percent of baseline (20 min preceding HFS). After recordings were completed, the electrodes were removed, the animal was transcardially perfused with 4% paraformaledhyde (PFA). The brain was dissected and immersed in 4% PFA over night at 4°C, then in 30% sucrose for 2 days at 4°C. Twenty micrometer coronal sections were cut using Tissue-Tek (Sakura), mounted on Superfrost GOLD slides (Braunschweig, Germany), and stored at 4°C.

### Immunofluorescence of Brain Sections

Antigen was retrieved by microwaving the mounted sections for 10 min at 600 W in citrate buffer (10 mM sodium citrate, 0.05% Tween-20, pH 6.0). After cooling for 20 min, sections were washed with PBS, permeabilized for 1.5 h with 0.5% Triton-X-100/PBS, washed with PBS and blocked with 5% horse serum/5% bovine serum albumin/PBS for 1 h at RT. Primary antibodies (Table 1) were diluted in blocking buffer, incubated overnight at 4°C, washed with PBS, then incubated with secondary antibodies (Table 1) for 1 h at RT, washed for 30 min, mounted, and coverslipped with ProLongGold Antifade Reagent containing DAPI (Invitrogen).

### Proximity Ligation Assay (PLA)

PLA was performed using the Duolink PLA Kit^1^ with red (DUO92008) or orange (DUO92007) detection reagents, anti-mouse minus probe (DUO92004), and anti-rabbit plus probe (DUO92002). Manufacturer’s instructions were followed for cultured neurons except that Roche blocking solution was used. For F-actin staining, phalloidin-FITC (Sigma; 0.5 μg/mL) was added in the penultimate wash step (wash buffer B) for 10 min. On brain sections, antigen retrieval was performed as above, all incubation times and wash steps were doubled, and the blocking buffer consisted of PBS containing 5% horse serum and 5% bovine serum albumin.

Images were taken on a Leica SP5 Laser Scanning confocal microscope. Immunofluorescence of cultured neurons was imaged with a 63× objective, a 561 nm laser for Alexa Fluor 568, a 633 laser for Alexa Fluor 647, and a 402 laser for DAPI. Two optical sections were imaged—one at the dendritic level and the other at the equatorial plane of the nucleus. Dendritic PLA and phalloidin-FITC staining were imaged using a 100× objective. For PLAs of cultured neurons, 24 z-stacks of 30 optical sections were taken from each coverslip using a 40× objective, 402 nm excitation for DAPI, and 598 nm excitation for the red PLA signal or 561 nm for the orange PLA. For PLA on brain sections, tile scans were taken at 40× of 5× 4 z-stacks of 14 optical sections, covering the DG.

Confocal stacks were analyzed with Imaris (Bitplane, RRID:SCR_007370) using the 3D “spot” function for PLA spots and “surface” function for nuclei. Optimal parameters for these functions were determined using the negative control (no antibodies) and positive control (Arc/Arc) from each experiment and kept constant for all images belonging to this experiment. In cell culture, PLA was quantified as the number of spots divided by the number of cells. In brain sections, the volumes of the molecular and granule cell layers (GCLs) were measured by creating a “surface object”, defined by the lateral boundaries of the layers in XY direction, and the tissue surface in Z direction. PLA was quantified as spot density (PLA/mm^3^) inside this defined volume. All analyses were performed by investigators blind to the experimental conditions.

### Transferrin Uptake Assay

SH-SY5Y human neuroblastoma cells were plated on 1 cm coverslips coated with collagen at a concentration of 0.3 μg/mL and were maintained in complete DMEM. Cells were transiently transfected at 70% confluence using Lipofectamine 2000 (Invitrogen) and 2 μg of Arc-GFP, calnexin C-terminus-mCherry, or both. Transferrin uptake assays were performed 24 h after transfection. Cells were serum-starved for 6 h prior to experiments (DMEM + 0.5% FBS). Alexa Fluor 647-conjugated Transferrin (Life Technologies T-23366) was added to each sample to a final concentration of 15 μg/mL for 30 min on ice. The samples were then returned to 37°C for 15 min to restore endocytosis. Cells were immediately placed on ice, washed twice with cold PBS, and fixed using 1% PFA on ice for 30 min. Coverslips were mounted using Prolong Gold anti-fade reagent with DAPI.

All cells were imaged on a Leica SP2 confocal microscope using a 60× objective. Identical settings were used for each sample. Images were analyzed in ImageJ (RRID:SCR_003070). Endocytosis was quantified by integrating the background-subtracted Alexa Fluor 647 signal within the boundaries of each cell. Transfected cells within each sample, determined by the presence of GFP (Arc), mCherry (calnexin), or both, were compared to internal controls (untransfected cells from the same slide, in the same field of view), and conditions were compared to each other using ANOVA after first normalizing to internal controls for each replicate. All analyses were performed by investigators blind to the experimental conditions.

## Results

### Arc Interacts with Calnexin

**Figure 1:**
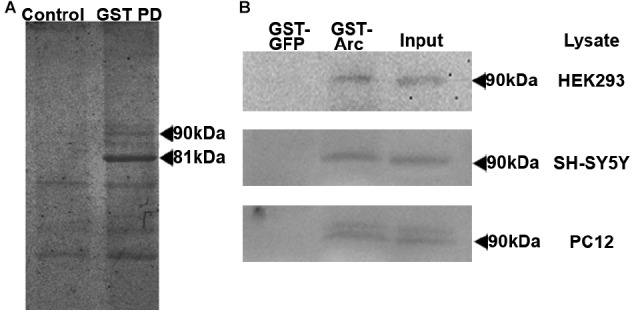
Activity-regulated cytoskeleton-associated protein (Arc) binds to calnexin in GST pull-downs. **(A)** GST-affinity purification was performed with HEK293 cell lysates and GST-Arc was used as bait. The prominent band at 81 kDa (right lane) was identified by mass spectrometry to be GST-Arc. The unidentified band at 90 kDa in the experimental lane and not in the control lane (containing GST, beads and lysate) was identified by mass spectrometry to be calnexin. **(B)** The pull-down was repeated with lysates from three different cell lines (HEK293, SH-SY5Y and PC12), transferred onto nitrocellulose membranes, and were probed for calnexin.

To identify novel Arc binding partners, we performed GST-affinity purification using recombinantly-expressed full-length rat Arc as bait (Arc-GST) and HEK293 cell lysate (Figure 1). The prominent band at 81 kDa on the Coomassie Brilliant Blue-stained SDS-PAGE gel was GST-Arc, as confirmed by MALDI-TOF mass spectrometry. The unidentified 90 kDa band seen in the experimental lane, but not in the control (GST), was excised from the gel and also analyzed by mass spectrometry (Figure 1A). The band was identified as a transmembrane protein of the endoplasmic reticulum, calnexin (Peptide Identification Score: 142; Peptides identified: APVPTGEVYFADSFDR and KIPNPDFFEDLEPFR). This finding was confirmed by probing GST-Arc-bound protein on a western blot with anti-calnexin antibody (Figure 1B, upper blot). GST-Arc also bound calnexin from non-stimulated SH-SY5Y neuroblastoma cells, a neuronal cell line in which Arc protein is endogenously expressed (Figure 1B, middle blot). Since both HEK293 and SH-SY5Y cells are of human origin, while our GST-Arc construct contained the rat sequence, we also performed the GST-pull-down with rat PC12 cells, a neuron-like cell line from the adrenal gland. Western blot analyses confirmed the presence of calnexin in each GST-Arc lane (Figure 1B).

### Calnexin Requires Arc Residues 200–225

**Figure 2:**
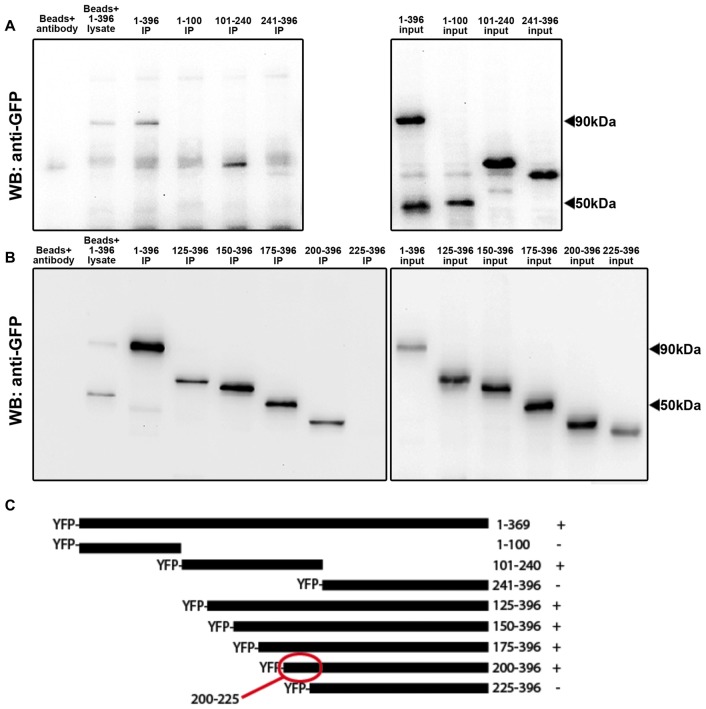
Arc residues 200–225 are required for the interaction with calnexin. Co-immunoprecipitations (IPs) were performed with a calnexin antibody and yellow fluorescent protein (YFP)-Arc deletion mutants. **(A)** Western blots probed with anti-GFP showed that full-length Arc (1–396) and Arc 101–240 bound to calnexin (left panel). Similar expression levels of YFP-Arc deletion mutants were confirmed by immunoblotting lysates with anti-GFP antibody (input, right panel). **(B)** To narrow down the binding region, co-IPs were repeated with progressive deletions of the Arc C-terminus. Arc 200–396 bound to calnexin while Arc 225–396 did not, indicating that Arc residues 200–225 are required for calnexin to interact (left panel). Equal loading was again ensured by immunoblotting equal amounts of lysates alone (input, right panel). **(C)** Alignment of the different Arc-deletion mutants. Numbers indicate amino acid positions. ± signs indicate positive or negative co-IPs with calnexin.

We then sought to determine the specific region of Arc protein that binds to calnexin. We constructed plasmids encoding the full-length rat Arc mRNA sequence, N-terminally fused to YFP-Arc (Figure 2C). Three deletion mutants of this construct, covering amino acids 1–100, 101–240 and 241–396, respectively, were ectopically expressed in HEK293 cells and subjected to IP with a calnexin-specific antibody. We found that YFP-Arc (101–240) co-immunoprecipitated with calnexin, while YFP-Arc (1–100) and YPF-Arc (241–396) failed to do so (Figure 2A, left panel). To further narrow down the binding region, we made sequential N-terminal deletions of YFP-Arc, expressing amino acids 125–396, 150–396, 175–396, 200–396 and 225–396, respectively. Of these fragments, only one failed to co-IP with calnexin, namely the shortest fragment YFP-Arc (225–396). Since YFP-Arc (200–396) is only 25 amino acids longer, but does co-IP with calnexin, Arc residues 200–225 are essential for the interaction with calnexin (Figure 2B, left panel).

### Arc Binds to the Calnexin C-Terminus in Hippocampal Neuronal Cultures

**Figure 3:**
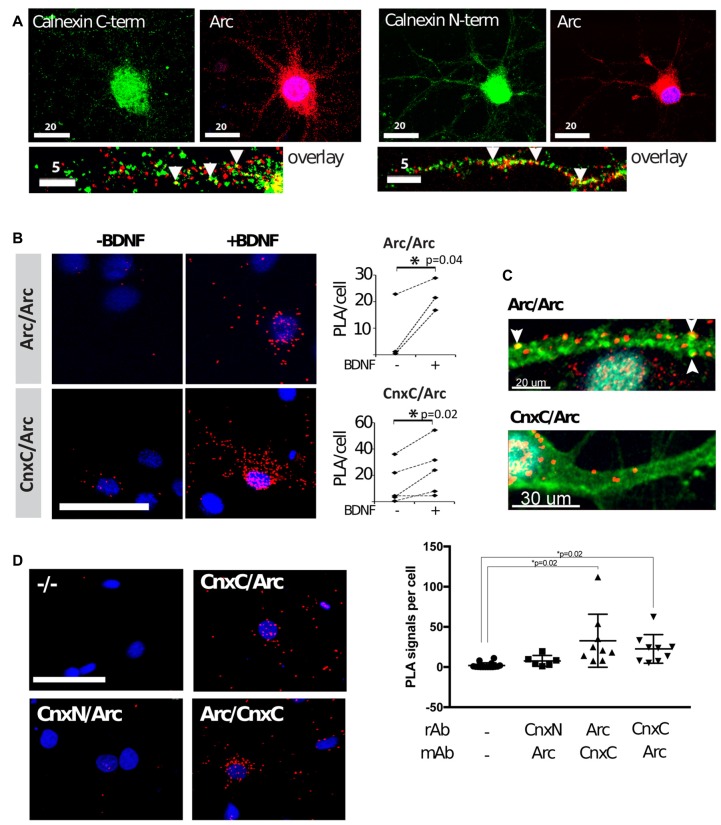
Arc binds to the calnexin C-terminus in hippocampal neuronal cultures. **(A)** Dual-immunofluorescence was performed with mouse anti-Arc C-7, rabbit anti-calnexin C-terminal, or rabbit anti-calnexin N-terminal, respectively, in brain-derived neurotrophic factor (BDNF)-treated neurons. Partial colocalization was observed between Arc and calnexin (both the N- and C-termini) in dendrites (arrowheads). Images are maximum intensity projections of two confocal sections: one at the dendritic level, and the other at the equatorial plane of the nucleus, taken with a 63× objective. **(B)** Proximity ligation assays (PLAs) were performed in neurons before and after BDNF treatment to confirm that Arc and calnexin are in close proximity (CnxC/Arc). Arc/Arc was used as a positive control. The images are maximum projections of the whole cells using a 40× objective. PLA of negative controls was subtracted from the experimental samples before statistical analyses (*N* = 3–4 independent experiments; 500–2, 200 cells/experiment; Wilcoxon matched-pairs signed rank test; Test statistics: *W* = 28, *p* = 0.016, z = 2.366). Dashed lines connect the mean PLA signals of treated and non-treated cells in each independent experiment. **(C)** PLA was again performed in BDNF-treated neurons. The F-actin stain phalloidin-FITC (green) was used in addition to PLA (red). PLA signals of Arc/Arc are abundant in the dendritic shaft and phalloidin-positive punctae (arrowheads). Arc/Cnx signals are also found in the shaft, but were less abundant and were never found in spines. A 63× objective was used, and images are maximum projections. Two independent experiments, about 100 cells each, were performed. **(D)** PLAs with Arc and calnexin antibodies recognizing the C-terminal (CnxC) and N-terminal (CnxN) were performed in BDNF-treated neurons. Images are maximum projections using a 40× objective. The negative control (−/−) did not contain primary antibodies. Right panel: values are expressed as mean ± SEM, where 700–2200 cells were analyzed per condition (Power analyses: CnxN-Arc = 0.695, Arc-CnxC = 0.793, CnxC-Arc = 0.658). Arc was found in close proximity to the C-terminal, but not the N-terminal.

We then examined whether the interaction between Arc and calnexin occurred in neurons. To induce Arc expression in primary hippocampal neurons, we stimulated the cells with brain-derived neurotrophic factor (BDNF) for 4 h. As expected, Arc was detected in the nucleus, neuronal soma and dendrites by immunofluorescence (Figure 3A). Since calnexin is a transmembrane protein, with the C-terminus located in the cytosol and the N-terminus located in the lumen of the endoplasmic reticulum, we used antibodies directed against either the C- or N-terminus. Both antibodies resulted in very similar staining patterns—a reticular cytoplasmic staining, including the nuclear membrane, and a weaker punctate dendritic pattern (Figure 3A). Partial colocalization was observed between Arc and both the calnexin C- and N-termini in dendrites (Figure 3A, arrowheads). This colocalization was rather sparse and merely indicates that a small fraction of both Arc and calnexin localize at or near the same sub-cellular site. To validate the interaction in neurons, we performed PLAs, which detect protein proximity below 40 nm. PLAs detect the proximity of two primary antibodies by labeling the interaction site with a fluorophore, which is then detected by fluorescence microscopy and quantified (Fredriksson et al., 2002).

We used well-characterized Arc-directed antibodies for the positive control, namely: (1) polyclonal rabbit anti-Arc, directed against full-length Arc (Synaptic Systems); and (2) monoclonal mouse anti-Arc (C-7, Santa Cruz). The probability of observing proximity between these antibodies is high, given that they bind to the same protein, thus forming intramolecular PLA. Expectedly, PLA signal for Arc/Arc was low in naïve neuronal cultures, in agreement with low basal Arc expression. After induction of Arc expression with BDNF for 4 h, Arc/Arc PLA was significantly upregulated (Figure 3B). Arc/Arc PLA punctae were frequently found at phalloidin-labeled dendritic punctae, most likely dendritic spines or synapses (Figure 3C). They were also located in the dendritic shaft and neuronal soma, but rarely in the nucleus. Arc/Arc PLA signal thus confidently reflects Arc expression and is suitable as a positive control.

To probe the interaction between Arc and calnexin, we used the same antibodies as for immunofluorescence (rabbit anti-calnexin C-terminus and mouse anti-Arc). This was important, since Arc is a cytoplasmic protein (lacking a signal peptide), and thus should only be able to interact with the calnexin C-terminus. PLA between calnexin and Arc was low in naïve neurons, and clearly positive in BDNF-treated neurons (Figure 3B). Thus, PLA confirmed the proximity between Arc and calnexin in cultured neurons. The Arc/calnexin C-terminus PLA was readily observed in neuronal soma and dendritic shaft, but was not in detected spines (Figure 3C).

We performed PLA with different combinations of antibodies to minimize the possibility of false-positive artifacts. PLA with rabbit anti-Arc and mouse anti-calnexin C-terminus gave the same result as the reverse above, which was significantly higher than the negative control in BDNF-treated neurons (Figure 3D). When using the rabbit anti-calnexin N-terminal antibody, PLA with Arc was not significantly different from the negative controls, although it seemed slightly elevated (Figure 3D). These results reflect the further distance between the lumenal N-terminus of calnexin and cytoplasmic Arc. Inconsistencies during sample preparation (most importantly, membrane permeabilization by Triton-X-100) could enhance or reduce this proximity.

### Arc Binds to the Calnexin C-Terminus *In Vivo*

**Figure 4:**
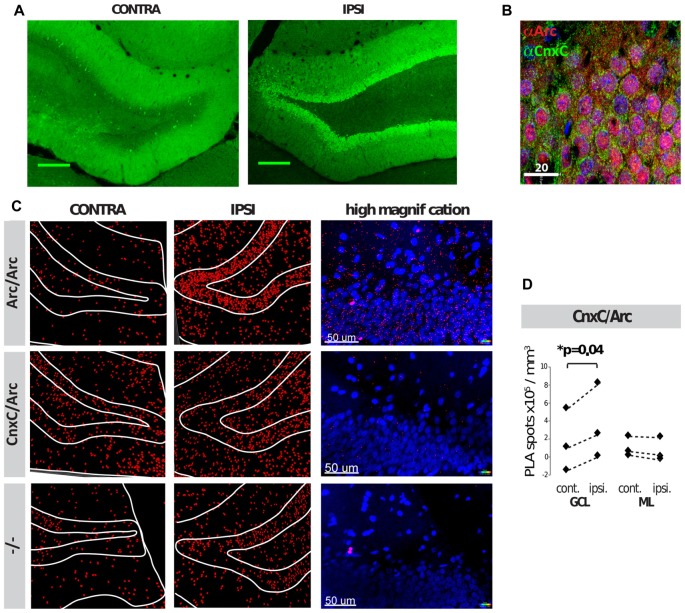
Arc and calnexin interact in rat brain sections following unilateral high-frequency stimulation (HFS). **(A)** Arc immunofluorescence of the contra- and ipsilateral dentate gyrus (DG) following HFS. **(B)** Dual-immunofluorescence of Arc (red), calnexin C-terminal (green) and DAPI (blue) of the ipsilateral (receiving HFS) DG, upper granule cell layer (GCL). **(C)** PLAs of HFS-treated tissue show Arc/Arc PLA signal (red) on both hemispheres, but Arc is robustly upregulated on the ipsilateral side. White lines outline the boundaries of the layers of the DG. **(D)** 3D quantification of CnxC/Arc PLA signal showed a significant increase in the granule cell layer (GCL), but not the molecular layer (ML) following HFS in the ipsilateral hemisphere (*p* = 0.04; *N* = 3 independent experiments; Power analyses: GCL = 0.973, ML = 0.838). Spots counted in the negative control (−/−) were subtracted prior to statistical analyses. Dashed lines connect the respective mean PLA signals of contra- and ipsi-lateral hemispheres in each independent experiment.

PLAs were also performed in coronal brain sections following HFS of the medial perforant path input to the DG of adult anesthetized rats. HFS induces robust upregulation of Arc in the granule cells of the DG, which is necessary for LTP maintenance (Steward et al., 1998; Messaoudi et al., 2007). Two hours after unilateral application of HFS, brains were transcardially fixed, embedded, cryosectioned and subjected to dual immunofluorescence or PLA. As expected, immunofluorescence showed that Arc was robustly upregulated in the DG granule cells of the ipsilateral hemisphere (Figure 4A), present both in nuclei and neuronal soma of the GCL (Figure 4B). Within the GCL, calnexin was seen in the perinuclear region of granule cell soma, but was excluded from the nuclei, as expected (Figure 4B). No obvious difference in calnexin expression was seen between the ipsilateral and contralateral brain hemispheres (data not shown). PLAs were then performed on neighboring sections of the same brains. PLA between Arc/Arc followed the same pattern as Arc expression—low in the contralateral hemisphere and high in the ipsilateral GCL (Figure 4C). PLA between calnexin and Arc is also significantly increased in the ipsilateral hemisphere, particularly in the GCL, but not the molecular layer (ML; Figures 4C,D). These assays demonstrate that the calnexin C-terminus and Arc interact in the DG GCL following HFS *in vivo*.

### Arc-Calnexin C-Terminus Interaction Is Not Involved in Endocytosis

**Figure 5:**
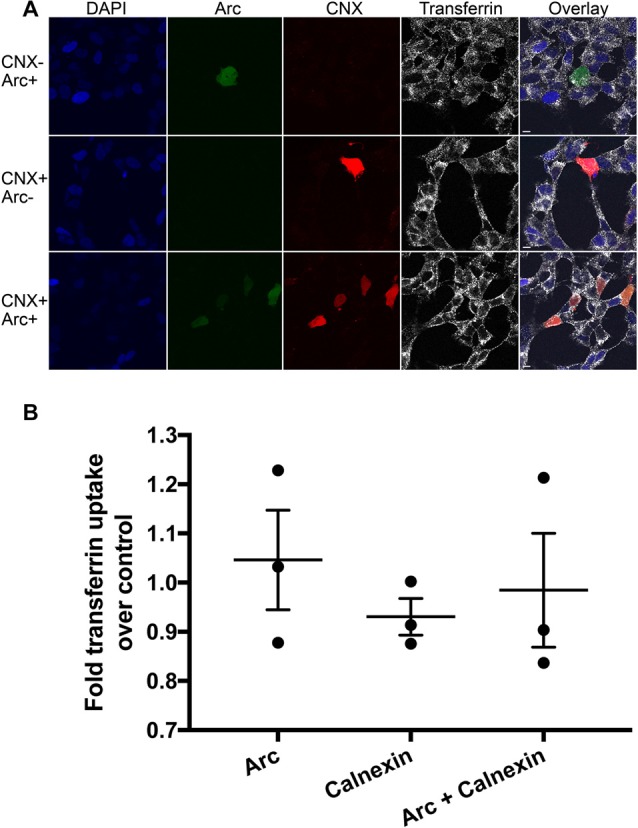
Receptor endocytosis assay. **(A)** Arc, calnexin, or both were overexpressed in SH-SY5Y neuroblastoma cells and the amount of endocytosed transferrin was quantified via confocal microscopy. **(B)** No significant difference in endocytosis was detected between the three groups (*p* = 0.25; Power = 0.864). Scale bars = 10 μm.

Lastly, we began to investigate the role of the Arc-calnexin interaction. Arc regulates endocytosis of AMPA receptors, where co-overexpression of Arc with endophilin increases AMPAR endocytosis (Chowdhury et al., 2006). Calnexin also regulates endocytosis, but overexpression of full-length calnexin or calnexin C-terminus impairs clathrin-mediated endocytosis (Li et al., 2011). We therefore examined whether the interaction affects receptor endocytosis by carrying out receptor uptake assays in neuroblastoma cells. The amount of endocytosed transferrin was quantified via confocal microscopy in three independent experiments (*n* = 63, 51 and 39 cells, respectively). No significant difference in endocytosis was detected between the Arc, calnexin, and Arc/calnexin groups (Figure 5; *p* = 0.246).

## Discussion

**Figure 6:**
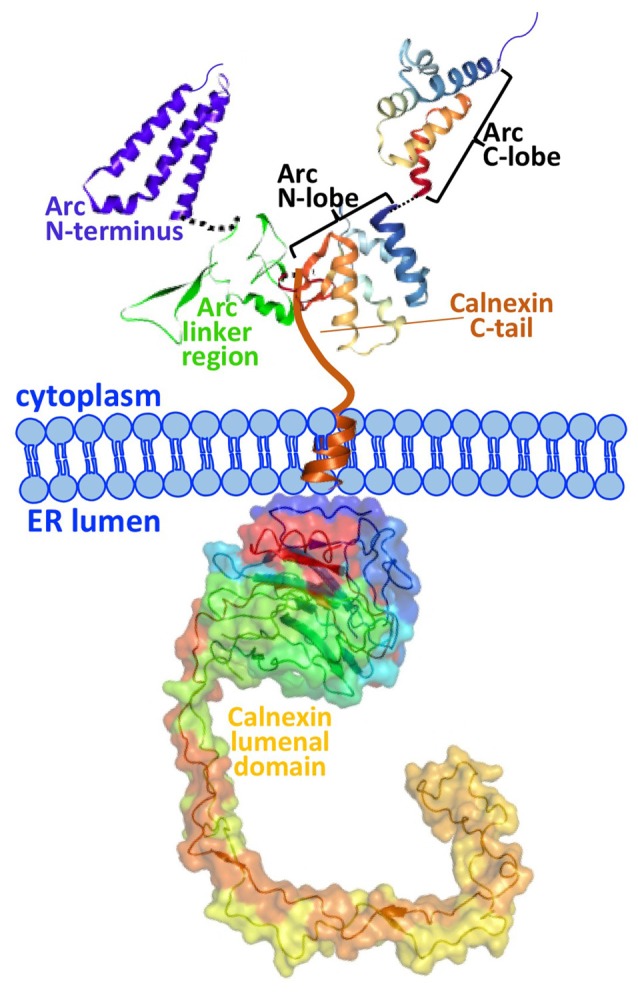
Schematic of the Arc-calnexin interaction. The C-terminal end of Arc’s central linker region interacts with the cytoplasmic C-terminus of calnexin in hippocampal neurons. The 3D structure from the Arc C-terminal domain is from Zhang et al. (2015). The predicted structure of the Arc N-terminus and linker region is from Myrum et al. (2015).

The present work identifies calnexin, a transmembrane protein of the ER, as a novel interaction partner of Arc. Calnexin is an ubiquitously-expressed protein best known for its role in the quality control of nascent glycoproteins in the ER (Caramelo and Parodi, 2008). We show that Arc binds to the C-terminal tail of calnexin in hippocampal neurons. We further show that Arc residues 200–225 are required for the interaction with calnexin (Figure 2). This region is at the border between the central flexible hinge region (~131–208) and the highly structured Arc C-terminal domain (residues ~209–365). A schematic of the Arc-calnexin interaction is shown in Figure 6.

BDNF treatment induces Arc expression and enhanced PLA signal with calnexin in neuronal somata and dendritic shafts. Although Arc was clearly detected in dendritic spines, no colocalization with calnexin was observed in spines (Figure 3C). In the DG of live rats, the Arc/calnexin PLA signal was significantly increased in the GCL at 2 h after LTP-inducing stimulation of the perforant path input. In the dendritic ML, the PLA signal was unchanged despite maximal Arc expression at this time (Messaoudi et al., 2007), suggesting that the primary interaction of Arc with calnexin occurs in granule cell somata (Figures 4C,D). It appears that Arc interacts with calnexin in the basal state, prior to experimental induction of Arc by BDNF, raising the possibility that Arc is stably associated with calnexin at the ER. This is consistent with an ultrastructural analysis showing a pool of immunoperoxidase-labeled Arc protein along ER membranes of granule cell somata and dendrites (Rodríguez et al., 2005).

The best described molecular role of Arc involves endocytosis, including homeostatic scaling (Shepherd et al., 2006), mGluR-LTD (Park et al., 2008) and its interaction with clathrin adaptor protein AP-2, dynamin-2 and endophilin 2/3 to selectively internalize AMPARs (Chowdhury et al., 2006; DaSilva et al., 2016). In contrast, the cytoplasmic C-tail of calnexin is a potent inhibitor of clathrin-mediated endocytosis (Li et al., 2011). We hypothesized that in a calnexin-bound state, Arc either promotes endocytosis by blocking the effect of the calnexin C-tail or inhibits endocytosis by blocking the interaction with the endocytic machinery. However, transferrin uptake assays showed no significant effects of calnexin/Arc overexpression compared to either calnexin or Arc alone (Figure 5). Since overexpression of Arc alone does not affect endocytosis rates in HeLa cells (Chowdhury et al., 2006), the interaction with calnexin may require another adaptor protein to affect endocytosis.

The region of calnexin binding is critical for multiple Arc protein-protein interactions and regulation. The C-terminal end of the putative linker region binds AP-2 and dynamin 2 (Shepherd et al., 2006; DaSilva et al., 2016), and ERK-catalyzed phosphorylation of S206 regulates the subcellular localization of Arc (Nikolaienko et al., 2017b). The adjacent Arc C-terminal domain is a bilobar structure in which the “N-lobe” region (207–278) is an interaction site for several proteins, including TARPγ2, αCaMKII, GKAP, WAVE1, IQSEC2 and GluN2A/B (Zhang et al., 2015). One of these proteins, TARPγ2, belongs to a family of AMPAR-binding auxiliary subunits intimately involved in AMPAR function including surface expression, synaptic targeting, as well as processing AMPARs in the ER and promoting their exit from the ER (Vandenberghe et al., 2005; Haering et al., 2014). The ER too is able to tune the spatial dimensions of AMPAR surface expression and accumulation at synapses (Ramírez and Couve, 2011; Cui-Wang et al., 2012), and calnexin itself binds several receptors, including AMPARs (Rubio and Wenthold, 1999). Arc’s interaction with both an inhibitor of endocytosis (calnexin) as well as endocytic machinery (dynamin-2, endophilin 2/3 and TARPγ2) points to Arc’s role as a molecular switch to regulate these processes.

In addition to calnexin’s role in receptor trafficking, it will be interesting to explore whether Arc is involved in the canonical functions of calnexin as a chaperone and regulator of protein quality control. It is also possible that the Arc/calnexin interaction is involved in calnexin’s less characterized pathways. For example, calnexin is actively recruited to the plasma membrane in an NMDAR-dependent manner, indicating that calnexin is involved in the trafficking of synaptic proteins to the plasma membrane (Itakura et al., 2013). Calnexin is continuously endocytosed, but it is sent to lysosomes for degradation instead to the pool of recycling endosomes (Okazaki et al., 2000). Evidence also suggests that the calnexin C-tail acts as a calcium sensor (Roderick et al., 2000), and such sensors are thought to regulate receptor trafficking to control synaptic strength (Turrigiano, 2008). Finally, calnexin directly interacts with sarco endoplasmic reticulum calcium ATPase (SERCA; Roderick et al., 2000), which sequesters cytosolic calcium back to the ER (Treiman et al., 1998), by which it determines ER calcium content (Lynes et al., 2013).

In summary, we establish here that: (1) Arc interacts with calnexin, an integral ER chaperone protein; (2) the region of Arc already known to be a binding hub also interacts with calnexin; (3) Arc binds to the small cytosolic domain of calnexin; (4) the Arc-calnexin association is significantly elevated following stimulation in hippocampal neuron cell bodies and dendritic shafts; (5) the Arc-calnexin association is significantly elevated in the dentate granule cell somata following *in vivo* HFS of the perforant path input; and (6) the interaction does not appear to play a role in receptor endocytosis.

## Ethics Statement

This study was approved by Norwegian National Research Ethics Committee in compliance with EU Directive 2010/63/EU, ARRIVE guidelines. Persons involved in the animal experiments have approved Federation of Laboratory and Animal Science Associations (FELASA) C course certificates and training.

## Author Contributions

CM, JS, MB and CRB designed research. CM, JS, MB, KC, KG, SKZ, MSE, RRN, SP and AS performed research. CM, MB and CRB wrote the article. All authors edited and approved the article.

## Conflict of Interest Statement

The authors declare that the research was conducted in the absence of any commercial or financial relationships that could be construed as a potential conflict of interest.

## Footnotes

^1^http://sigma.com/duolink

## References

[B1] Alberi L. Liu S. Wang Y. Badie R. Smith-Hicks C. Wu J.. (2011). Activity-induced Notch signaling in neurons requires Arc/Arg3.1 and is essential for synaptic plasticity in hippocampal networks. Neuron 69, 437–444. 10.1016/j.neuron.2011.01.004 21315255 PMC3056341

[B2] Bloomer W. A. C. VanDongen H. M. A. VanDongen A. M. J. (2007). Activity-regulated cytoskeleton-associated protein Arc/Arg3.1 binds to spectrin and associates with nuclear promyelocytic leukemia (PML) bodies. Brain Res. 1153, 20–33. 10.1016/j.brainres.2007.03.079 17466953

[B3] Bloomer W. A. C. VanDongen H. M. A. VanDongen A. M. J. (2008). Arc/Arg3.1 translation is controlled by convergent *N*-methyl-D-aspartate and Gs-coupled receptor signaling pathways. J. Biol. Chem. 283, 582–592. 10.1074/jbc.M702451200 17981809

[B4] Bramham C. R. Alme M. N. Bittins M. Kuipers S. D. Nair R. R. Pai B.. (2010). The Arc of synaptic memory. Exp. Brain Res. 200, 125–140. 10.1007/s00221-009-1959-2 19690847 PMC2803749

[B5] Byers C. E. Barylko B. Ross J. A. Southworth D. R. James N. G. Taylor C. A. IV. (2015). Enhancement of dynamin polymerization and GTPase activity by Arc/Arg3.1. Biochim. Biophys. Acta 1850, 1310–1318. 10.1016/j.bbagen.2015.03.002 25783003 PMC4398645

[B6] Caramelo J. J. Parodi A. J. (2008). Getting in and out from calnexin/calreticulin cycles. J. Biol. Chem. 283, 10221–10225. 10.1074/jbc.R700048200 18303019 PMC2447651

[B7] Chowdhury S. Shepherd J. D. Okuno H. Lyford G. Petralia R. S. Plath N.. (2006). Arc/Arg3.1 interacts with the endocytic machinery to regulate AMPA receptor trafficking. Neuron 52, 445–459. 10.1016/j.neuron.2006.08.033 17088211 PMC1784006

[B8] Craig T. J. Jaafari N. Petrovic M. M. Rubin P. P. Mellor J. R. Henley J. M. (2012). Homeostatic synaptic scaling is regulated by protein SUMOylation. J. Biol. Chem. 287, 22781–22788. 10.1074/jbc.M112.356337 22582390 PMC3391081

[B9] Cui-Wang T. Hanus C. Cui T. Helton T. Bourne J. Watson D.. (2012). Local zones of endoplasmic reticulum complexity confine cargo in neuronal dendrites. Cell 148, 309–321. 10.1016/j.cell.2011.11.056 22265418 PMC3266556

[B10] DaSilva L. L. P. Wall M. J. de Almeida L. P. Wauters S. C. Januario Y. C. Mu ller J.. (2016). Activity-regulated cytoskeleton-associated protein controls AMPAR endocytosis through a direct interaction with clathrin-adaptor protein 2. eNeuro 3:ENEURO.0144-15.2016. 10.1523/ENEURO.0144-15.2016 27257628 PMC4877669

[B11] Fredriksson S. Gullberg M. Jarvius J. Olsson C. Pietras K. Gústafsdóttir S. M.. (2002). Protein detection using proximity-dependent DNA ligation assays. Nat. Biotechnol. 20, 473–477. 10.1038/nbt0502-473 11981560

[B12] Giorgi C. Yeo G. W. Stone M. E. Katz D. B. Burge C. Turrigiano G.. (2007). The EJC factor eIF4AIII modulates synaptic strength and neuronal protein expression. Cell 130, 179–191. 10.1016/j.cell.2007.05.028 17632064

[B13] Gozdz A. Nikolaienko O. Urbanska M. Cymerman I. A. Sitkiewicz E. Blazejczyk M.. (2017). GSK3α and GSK3β phosphorylate Arc and regulate its degradation. Front. Mol. Neurosci. 10:192. 10.3389/fnmol.2017.00192 28670266 PMC5472658

[B14] Greer P. L. Hanayama R. Bloodgood B. L. Mardinly A. R. Lipton D. M. Flavell S. W.. (2010). The angelman syndrome protein Ube3A regulates synapse development by ubiquitinating arc. Cell 140, 704–716. 10.1016/j.cell.2010.01.026 20211139 PMC2843143

[B15] Haering S. C. Tapken D. Pahl S. Hollmann M. (2014). Auxiliary subunits: shepherding AMPA receptors to the plasma membrane. Membranes (Basel) 4, 469–490. 10.3390/membranes4030469 25110960 PMC4194045

[B16] Itakura M. Tsujimura J. Yamamori S. Ohkido T. Takahashi M. (2013). NMDA receptor-dependent recruitment of calnexin to the neuronal plasma membrane. Neurosci. Lett. 550, 173–178. 10.1016/j.neulet.2013.06.064 23851254

[B17] Korb E. Finkbeiner S. (2011). Arc in synaptic plasticity: from gene to behavior. Trends Neurosci. 34, 591–598. 10.1016/j.tins.2011.08.007 21963089 PMC3207967

[B18] Korb E. Wilkinson C. L. Delgado R. N. Lovero K. L. Finkbeiner S. (2013). Arc in the nucleus regulates PML-dependent GluA1 transcription and homeostatic plasticity. Nat. Neurosci. 16, 874–883. 10.1038/nn.3429 23749147 PMC3703835

[B19] Li H.-D. Liu W.-X. Michalak M. (2011). Enhanced clathrin-dependent endocytosis in the absence of calnexin. PLoS One 6:e21678. 10.1371/journal.pone.0021678 21747946 PMC3128601

[B20] Lynes E. M. Raturi A. Shenkman M. Ortiz Sandoval C. Yap M. C. Wu J.. (2013). Palmitoylation is the switch that assigns calnexin to quality control or ER Ca^2+^ signaling. J. Cell Sci. 126, 3893–3903. 10.1242/jcs.125856 23843619

[B21] Mabb A. M. Je H. S. Wall M. J. Robinson C. G. Larsen R. S. Qiang Y.. (2014). Triad3A regulates synaptic strength by ubiquitination of Arc. Neuron 82, 1299–1316. 10.1016/j.neuron.2014.05.016 24945773 PMC4277707

[B22] Messaoudi E. Kanhema T. Soulé J. Tiron A. Dagyte G. da Silva B.. (2007). Sustained Arc/Arg3.1 synthesis controls long-term potentiation consolidation through regulation of local actin polymerization in the dentate gyrus *in vivo*. J. Neurosci. 27, 10445–10455. 10.1523/JNEUROSCI.2883-07.2007 17898216 PMC6673172

[B23] Minatohara K. Akiyoshi M. Okuno H. (2016). Role of immediate-early genes in synaptic plasticity and neuronal ensembles underlying the memory trace. Front. Mol. Neurosci. 8:78. 10.3389/fnmol.2015.00078 26778955 PMC4700275

[B24] Myrum C. Baumann A. Bustad H. Flydal M. Mariaule V. Alvira S.. (2015). Arc is a flexible modular protein capable of reversible self-oligomerization. Biochem. J. 468, 145–158. 10.1042/BJ20141446 25748042 PMC4422259

[B25] Nair R. R. Patil S. Tiron A. Kanhema T. Panja D. Schiro L.. (2017). Dynamic Arc SUMOylation and selective interaction with F-actin-binding protein drebrin A in LTP consolidation *in vivo*. Front. Synaptic Neurosci. 9:8. 10.3389/fnsyn.2017.00008 28553222 PMC5426369

[B26] Nikolaienko O. Eriksen M. S. Patil S. Bito H. Bramham C. (2017a). Stimulus evoked ERK-dependent phosphorylation of activity-regulated cytoskeleton-associated protein (Arc) regulates its neuronal subcellular localization. Neuroscience 360, 68–80. 10.1016/j.neuroscience.2017.07.026 28736134

[B70] Nikolaienko O. Pati S. Eriksen M. S. Bramham C. R. (2017b). Arc protein: a flexible hub for synaptic plasticity and cognition. Semin. Cell. Dev. Biol. [Epub ahead of print]. 10.1016/j.semcdb.2017.09.006 28890419

[B27] Okazaki Y. Ohno H. Takase K. Ochiai T. Saito T. (2000). Cell surface expression of calnexin, a molecular chaperone in the endoplasmic reticulum. J. Biol. Chem. 275, 35751–35758. 10.1074/jbc.M007476200 10956670

[B28] Panja D. Bramham C. R. (2014). BDNF mechanisms in late LTP formation: a synthesis and breakdown. Neuropharmacology 76, 664–676. 10.1016/j.neuropharm.2013.06.024 23831365

[B29] Panja D. Dagyte G. Bidinosti M. Wibrand K. Kristiansen A.-M. Sonenberg N.. (2009). Novel translational control in Arc-dependent long term potentiation consolidation *in vivo*. J. Biol. Chem. 284, 31498–31511. 10.1074/jbc.M109.056077 19755425 PMC2797219

[B30] Park S. Park J. M. Kim S. Kim J.-A. Shepherd J. D. Smith-Hicks C. L.. (2008). Elongation factor 2 and fragile X mental retardation protein control the dynamic translation of Arc/Arg3.1 essential for mGluR-LTD. Neuron 59, 70–83. 10.1016/j.neuron.2008.05.023 18614030 PMC2743934

[B31] Peebles C. L. Yoo J. Thwin M. T. Palop J. J. Noebels J. L. Finkbeiner S. (2010). Arc regulates spine morphology and maintains network stability *in vivo*. Proc. Natl. Acad. Sci. U S A 107, 18173–18178. 10.1073/pnas.1006546107 20921410 PMC2964216

[B32] Plath N. Ohana O. Dammermann B. Errington M. L. Schmitz D. Gross C.. (2006). Arc/Arg3.1 is essential for the consolidation of synaptic plasticity and memories. Neuron 52, 437–444. 10.1016/j.neuron.2006.08.024 17088210

[B33] Ramírez O. A. Couve A. (2011). The endoplasmic reticulum and protein trafficking in dendrites and axons. Trends Cell Biol. 21, 219–227. 10.1016/j.tcb.2010.12.003 21215635

[B34] Roderick H. L. Lechleiter J. D. Camacho P. (2000). Cytosolic phosphorylation of calnexin controls intracellular Ca^2+^ oscillations via an interaction with SERCA2b. J. Cell Biol. 149, 1235–1247. 10.1083/jcb.149.6.1235 10851021 PMC2175122

[B35] Rodríguez J. J. Davies H. A. Silva A. T. De Souza I. E. J. Peddie C. J. Colyer F. M.. (2005). Long-term potentiation in the rat dentate gyrus is associated with enhanced Arc/Arg3.1 protein expression in spines, dendrites and glia. Eur. J. Neurosci. 21, 2384–2396. 10.1111/j.1460-9568.2005.04422.x 15932597

[B36] Rubio M. E. Wenthold R. J. (1999). Calnexin and the immunoglobulin binding protein (BiP) coimmunoprecipitate with AMPA receptors. J. Neurochem. 73, 942–948. 10.1046/j.1471-4159.1999.0730942.x 10461883

[B37] Shepherd J. D. Bear M. F. (2011). New views of Arc, a master regulator of synaptic plasticity. Nat. Neurosci. 14, 279–284. 10.1038/nn.2708 21278731 PMC8040377

[B38] Shepherd J. D. Rumbaugh G. Wu J. Chowdhury S. Plath N. Kuhl D.. (2006). Arc/Arg3.1 mediates homeostatic synaptic scaling of AMPA receptors. Neuron 52, 475–484. 10.1016/j.neuron.2006.08.034 17088213 PMC1764219

[B39] Soulé J. Alme M. Myrum C. Schubert M. Kanhema T. Bramham C. R. (2012). Balancing Arc synthesis, mRNA decay, and proteasomal degradation: maximal protein expression triggered by rapid eye movement sleep-like bursts of muscarinic cholinergic receptor stimulation. J. Biol. Chem. 287, 22354–22366. 10.1074/jbc.M112.376491 22584581 PMC3381195

[B40] Steward O. Wallace C. S. Lyford G. L. Worley P. F. (1998). Synaptic activation causes the mRNA for the IEG Arc to localize selectively near activated postsynaptic sites on dendrites. Neuron 21, 741–751. 10.1016/s0896-6273(00)80591-7 9808461

[B41] Treiman M. Caspersen C. Christensen S. B. (1998). A tool coming of age: thapsigargin as an inhibitor of sarco- endoplasmic reticulum Ca^2+^-ATPases. Trends Pharmacol. Sci. 19, 131–135. 10.1016/s0165-6147(98)01184-5 9612087

[B42] Turrigiano G. G. (2008). The self-tuning neuron: synaptic scaling of excitatory synapses. Cell 135, 422–435. 10.1016/j.cell.2008.10.008 18984155 PMC2834419

[B43] Vandenberghe W. Nicoll R. A. Bredt D. S. (2005). Interaction with the unfolded protein response reveals a role for stargazin in biosynthetic AMPA receptor transport. J. Neurosci. 25, 1095–1102. 10.1523/JNEUROSCI.3568-04.2005 15689545 PMC6725949

[B44] Waung M. W. Pfeiffer B. E. Nosyreva E. D. Ronesi J. A. Huber K. M. (2008). Rapid translation of Arc/Arg3.1 selectively mediates mGluR-dependent LTD through persistent increases in AMPAR endocytosis rate. Neuron 59, 84–97. 10.1016/j.neuron.2008.05.014 18614031 PMC2580055

[B45] Wu J. Petralia R. S. Kurushima H. Patel H. Jung M. Volk L.. (2011). Arc/Arg3.1 regulates an endosomal pathway essential for activity-dependent β-amyloid generation. Cell 147, 615–628. 10.1016/j.cell.2011.09.036 22036569 PMC3207263

[B46] Zhang W. Wu J. Ward M. D. Yang S. Chuang Y.-A. Xiao M.. (2015). Structural basis of arc binding to synaptic proteins: implications for cognitive disease. Neuron 86, 490–500. 10.1016/j.neuron.2015.03.030 25864631 PMC4409568

